# Rheumatoid arthritis and the risk of end-stage renal disease: A nationwide, population-based study

**DOI:** 10.3389/fmed.2023.1116489

**Published:** 2023-02-02

**Authors:** Sang Heon Suh, Jin Hyung Jung, Tae Ryom Oh, Eun Mi Yang, Hong Sang Choi, Chang Seong Kim, Eun Hui Bae, Seong Kwon Ma, Kyung-Do Han, Soo Wan Kim

**Affiliations:** ^1^Department of Internal Medicine, Chonnam National University Medical School and Chonnam National University Hospital, Gwangju, Republic of Korea; ^2^Department of Biostatistics, College of Medicine, Catholic University of Korea, Seoul, Republic of Korea; ^3^Department of Pediatrics, Chonnam National University Medical School, Chonnam National University Hospital, Gwangju, Republic of Korea; ^4^Department of Statistics and Actuarial Science, Soongsil University, Seoul, Republic of Korea

**Keywords:** rheumatoid arthritis, disease-modifying anti-rheumatic drugs, end-stage renal disease, chronic kidney disease, nation-wide population-based study

## Abstract

**Introduction:**

Despite the risk of incident chronic kidney disease among the patients with rheumatoid arthritis (RA), the association of RA and the risk of end-stage renal disease (ESRD) has not been clearly elucidated. We aimed to investigate the association of RA and the risk of ESRD.

**Materials and methods:**

A total of 929,982 subjects with (*n* = 154,997) or without (*n* = 774,985) RA from the National Health Insurance Service (NHIS) database in Koreas (corresponding to the period between 2009 and 2017) were retrospectively analyzed. RA was defined by the International Classification of Diseases, 10th Revision, Clinical Modification (ICD-10-CM), codes plus any dispensing of disease-modifying anti-rheumatic drugs. The primary outcome was incident ESRD, identified by a combination of the ICD-10-CM codes and a special code assigned to patients receiving maintenance dialysis for ≥ 3 months or those with a transplant kidney.

**Results:**

Compared to the subjects without RA, the subjects with RA resulted in an increased incidence of ESRD (incidence rates of 0.374 versus 0.810 cases per 1,000 person-years). Accordingly, compared to the subjects without RA, the risk of ESRD was significantly increased among the subjects with RA (adjusted hazard ratio 2.095, 95% confidence interval 1.902–2.308). Subgroup analyses revealed that the risk of ESRD imposed by RA is relatively higher in relatively young and healthy individuals.

**Conclusion:**

Rheumatoid arthritis (RA) increase the risk of ESRD. As the risk of ESRD imposed by RA is relatively higher in relatively young and healthy individuals, kidney-protective treatment, such as biologic agents, should be preferentially considered among these patients with RA.

## 1. Introduction

Chronic kidney disease (CKD) imposes a great burden on public health management system, with an estimated prevalence of 8–16% globally (1). A proportion of CKD progresses to end-stage renal disease (ESRD), the prevalence and incidence of which significantly varies regionally. In Korea, approximately 100,000 patients are treated with renal replacement therapy, and the number of patients with ESRD is rapidly growing recently (2). Hence, the identification of risk factors for the development and progression of CKD is becoming an issue of special concern.

Rheumatoid arthritis (RA) is a common chronic inflammatory disorder of the synovial joints and may eventually lead to permanent destruction of the involved joints unless it is adequately treated (3, 4). Due to the complexity of the disease, the current understanding of the pathophysiology and therapeutics for RA is still limited. For instance, although a large number of studies have shown that metformin treatment can reduce inflammation, delay disease progression and protect bone tissue in the RA progression, few studies focus on the relationship between metformin treatment and the risk of RA (5). Epidemiologic studies suggested that RA contributes to the other comorbidities, such as cardiovascular diseases (6–8). *In vivo* and *in vitro* studies demonstrated that oxidative stress and inflammatory cytokines triggered by chronic inflammatory process result in accelerated atherosclerosis in vascular beds (9, 10). In this context, RA may potentially increase the risk of development and progression of CKD due to the accelerated atherosclerosis. Higher prevalence of diabetes mellitus (DM) and hypertension (HTN) among the patients with RA has been also previously reported, which are well-known risk factors of CKD (11). Case reports of secondary glomerular lesions secondary to RA, such as mesangial proliferative glomerulonephritis and membranous nephropathy, have been published (12, 13). Further, medications chronically used in the management of RA, including biological and non-biological disease-modifying anti-rheumatic drugs (DMARDs) or analgesics like non-steroidal anti-inflammatory drugs (NSAIDs), may be directly detrimental to kidney function (14–16). Indeed, studies so far indicate that the risk of incident CKD is increased among the patients with RA (17, 18), despite some debates (19, 20). Yet, the association of RA and the risk of ESRD has not been clearly elucidated.

In the present study, we hypothesized that RA may increase the risk of ESRD. Taking advantage of a nationwide data from Nation Health Insurance Service (NHIS), we analyzed more than 154,997 patients with RA and age- and sex-matched 774,985 control subjects to determine RA as an independent risk factor of ESRD. We also conducted a series of subgroup analyses to define specific populations with higher risk of ESRD among the patients with RA.

## 2. Materials and methods

### 2.1. National Health Insurance Service data source

National Health Insurance Sharing Service provides publicly available anonymized data.^1^ In the present study, we used the national health insurance claims database established by the Korean NHIS, which includes sociodemographic data and all medical expenses for inpatient and outpatient services, pharmacy dispensing claims, and mortality data (21, 22). All insured Korean individuals older than 40 years of age undergo a biannual health checkup supported by the NHIS, and employed Koreans older than 20 years are required to undergo an annual health checkup (21). A subset of NHIS health checkup data corresponding to the period between 2009 and 2017 was analyzed in the current study. The institutional review board of Chonnam National University Hospital approved the study protocol (CNUH-EXP-2021-431). Patient identification numbers were anonymized to protect individual privacy. As all data to identify the individual patients were anonymized and deidentified for analysis, the institutional review board waived the need for informed consent.

### 2.2. Study population

We identified participants who were newly diagnosed with RA using the International Classification of Diseases, tenth Revision, Clinical Modification (ICD-10-CM), codes (M05.x, M06.x) plus any dispensing of DMARDs during the identification period from January 2010 to December 2017 (*n* = 286,148; 23). Disease-modifying anti-rheumatic drugs (DMARDs) in the current study included all of conventional synthetic DMARDs (e.g., methotrexate, hydroxychloroquine, leflunomide, sulfasalazine, tacrolimus, cyclosporine, D-penicillamine, bucillamine, and azathioprine), biological DMARDs (e.g., adalimumab, etanercept, infliximab, golimumab, rituximab, abatacept, and tocilizumab), and targeted synthetic DMARD (e.g., tofacitinib). Those who had not completed a health checkup within 2 years before the diagnosis of RA, those with any missing data, those with age < 20 years, and those with a history of ESRD before the diagnosis of RA were excluded. We additionally excluded the subjects who were diagnosed with ESRD or who died within 1 year after the diagnosis of RA, for the diagnostic accuracy of RA. For comparison between the subjects with and without RA, we selected the control group (without RA) by age-sex exact matching, and included five times as many subjects as RA cohort. Patients with RA who were not matched with controls without RA were further excluded.

### 2.3. Study design

**Figure 1:**
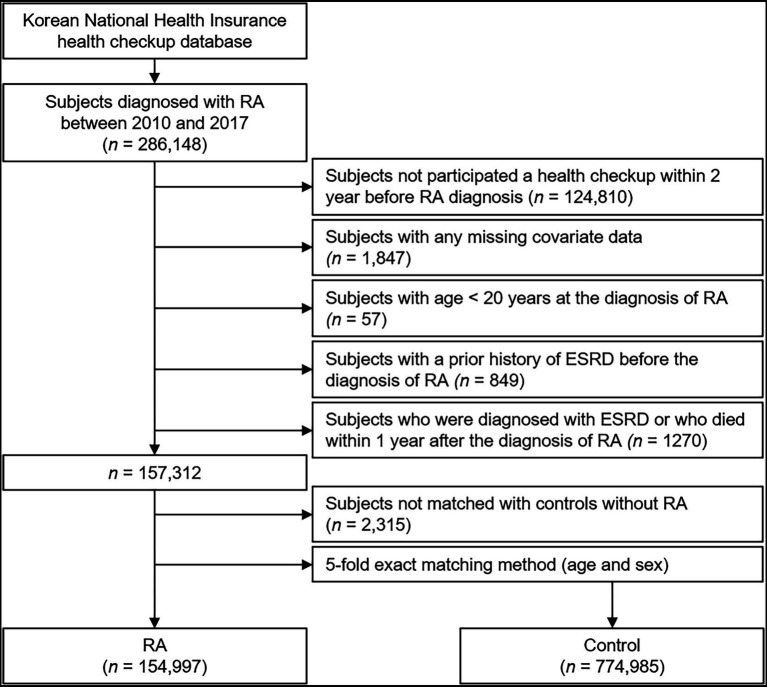
Flow diagram of the study population. ESRD, end-stage renal disease; RA, rheumatoid arthritis.

A total of 929,982 subjects with (*n* = 154,997) or without (*n* = 774,985) RA were followed up from baseline to the date of ESRD diagnosis, the date of death, or the last checkup before 31 December 2019. The median follow-up duration was 4.69 years (Figure 1).

### 2.4. Data collection

The details of the data collection were previously described (21). Briefly, information on the subject’s smoking status, alcohol consumption, body mass index (BMI), and waist circumference was obtained during health examinations. Obesity was defined as BMI ≥ 25 kg/m^2^ (24). Participation in regular exercise was determined by the response to the question, “Did you do mid-term exercise for > 30 min on more than 5 days or vigorous exercise for > 20 min on more than 3 days during the past week?.” The participants were divided according to income into quintiles, where a low income was defined as the first quintile. Comorbid conditions, such as HTN, DM, and DL, were defined as previously described (21). The history of NSAID medication was identified by the WHO ATC code (M01A) within the 1 year before index date. Estimated glomerular filtration rate (eGFR) was calculated using Modification of Diet in Renal Disease equation (25). CKD was defined as an eGFR < 60 ml/min/1.73m^2^.

### 2.5. Study outcomes

The study endpoint was incident ESRD, which was defined as a status of requiring hemodialysis, peritoneal dialysis, or kidney transplant (21). Patients with ESRD were identified by a combination of the ICD-10-CM codes (N18-N19, Z49, Z94.0, and Z99.2) and a special code assigned to patients receiving maintenance hemodialysis or peritoneal dialysis for ≥ 3 months or those with a transplant kidney (V001, procedure-related outpatient care or inpatient treatment on the day of hemodialysis; V003, peritoneal dialysis; and V005, kidney transplant; 21). Individuals who had a kidney transplant or dialysis code on the same date as an acute kidney failure code (N17.9) were excluded from the study outcome event. We also excluded individuals receiving continuous kidney replacement therapy or short-term peritoneal dialysis from the study outcome event.

### 2.6. Statistical analyses

Data were presented as means ± standard deviation for continuous variables and as the number and proportion for categorical variables. To compare the characteristics of interest between groups, 2-sample independent *t*-tests were applied to continuous variables, and Chi-square test was used to assess binary and categorical variables. The event rate was calculated per 1,000 person-years. To identify the risk of ESRD by RA, Cox proportional hazard regression models were analyzed with the adjustments for the potential confounding factors. Cox proportional hazard models were presented as hazard ratios (HRs) and 95% confidence intervals (CIs). Pre-specified subgroup analyses were conducted, where interaction terms were added to test for effect modification across subgroups. To validate our findings, we performed sensitivity analyses. First, to assess the possibility of reverse causation, we excluded the subjects with ESRD occurring within 3 and 5 years of follow-up. Second, because a mortality event could compete with our outcome of interest, we used cause-specific hazard models, in which the death occurring before reaching the primary outcome was treated as a competing risk and censored (26, 27). Finally, we adopted the propensity score-matching analysis to balance the differences in covariates. All statistical tests were two-tailed, and *p* values < 0.05 were considered statistically significant. All data analyses were conducted using SAS software (version 9.4; SAS Institute).

## 3. Results

### 3.1. Baseline characteristics of the study population

The baseline characteristics of the subjects with RA and their age- and sex-matched control subjects (without RA) is described in Table 1. Smoking rate and the proportion of those with alcohol consumption, regular exercise, and low-income status were higher among the control subjects. Whereas the proportion of those with obesity was higher in the control subjects, the prevalence of DM, HTN, and DL was higher in the subjects with RA. The prevalence of CKD at the baseline was not significantly different between the two groups. BMI, SBP, and DBP were lower in the subjects with RA. Fasting glucose and total cholesterol were higher in the control subjects. eGFR was higher in the subjects with RA at the baseline. To summarize, despite the substantial differences in the baseline characteristics between the two groups, the prevalence of CKD at the baseline was similar.

**Table 1:** Baseline characteristics of study population.

	Control	RA	*p* Value
Follow-up duration (years)	4.73 ± 2.23	4.70 ± 2.23	< 0.0001
Incidence of ESRD (%)	1,370 (0.18)	590 (0.38)	< 0.0001
Demographic information			
Age (year)	55.82 ± 11.9	55.82 ± 11.9	1
20–39	65,640 (8.47)	13,128 (8.47)	1
40–64	522,990 (67.48)	104,598 (67.48)	
65 ≤	186,355 (24.05)	37,271 (24.05)	
Male sex	207,740 (26.81)	41,548 (26.81)	1
Current smoking	93,430 (12.06)	18,194 (11.74)	0.0004
Alcohol consumption	248,322 (32.04)	45,824 (29.56)	< 0.0001
Regular exercise	154,182 (19.89)	28,627 (18.47)	< 0.0001
Low-income status	159,132 (20.53)	31,309 (20.2)	0.0029
Comorbid conditions			
Use of NSAIDs	603,804 (77.90)	151,966 (98.04)	< 0.001
Obesity	260,933 (33.67)	51,653 (33.33)	0.0088
DM	94,830 (12.24)	19,471 (12.56)	0.0004
HTN	275,821 (35.59)	58,709 (37.88)	< 0.0001
DL	241,597 (31.17)	52,411 (33.81)	< 0.0001
CKD	44,774 (5.78)	9,124 (5.89)	0.0931
Anthropometric measures			
Height (cm)	159.19 ± 8.36	159.34 ± 8.35	< 0.0001
Body weight (kg)	60.63 ± 10.48	60.58 ± 10.44	0.0987
BMI (kg/m^2^)	23.86 ± 3.25	23.8 ± 3.24	< 0.0001
Waist circumference (cm)	79.72 ± 9.08	79.74 ± 9.16	0.3012
SBP (mmHg)	122.73 ± 15.32	122.06 ± 15.06	< 0.0001
DBP (mmHg)	75.87 ± 9.98	75.5 ± 9.8	< 0.0001
Laboratory findings			
Fasting glucose (mg/dL)	99.54 ± 24.04	98.27 ± 23.01	< 0.0001
Total cholesterol (mg/dL)	198.71 ± 38.01	196.24 ± 38.98	< 0.0001
eGFR (mL/min./1.73m^2^)	89.4 ± 37.91	90.11 ± 36.99	< 0.0001

Values for categorical variables are given as number (percentage); values for continuous variables, as mean ± standard deviation.

BMI, body mass index; CKD, chronic kidney disease; DBP, diastolic blood pressure; DL, dyslipidemia; DM, diabetes mellitus; eGFR, estimated glomerular filtration rate; ESRD, end-stage renal disease; HTN, hypertension; NSAIDs, non-steroidal anti-inflammatory drugs; SBP, systolic blood pressure.

### 3.2. Association of RA with the risk of ESRD

**Figure 2:**
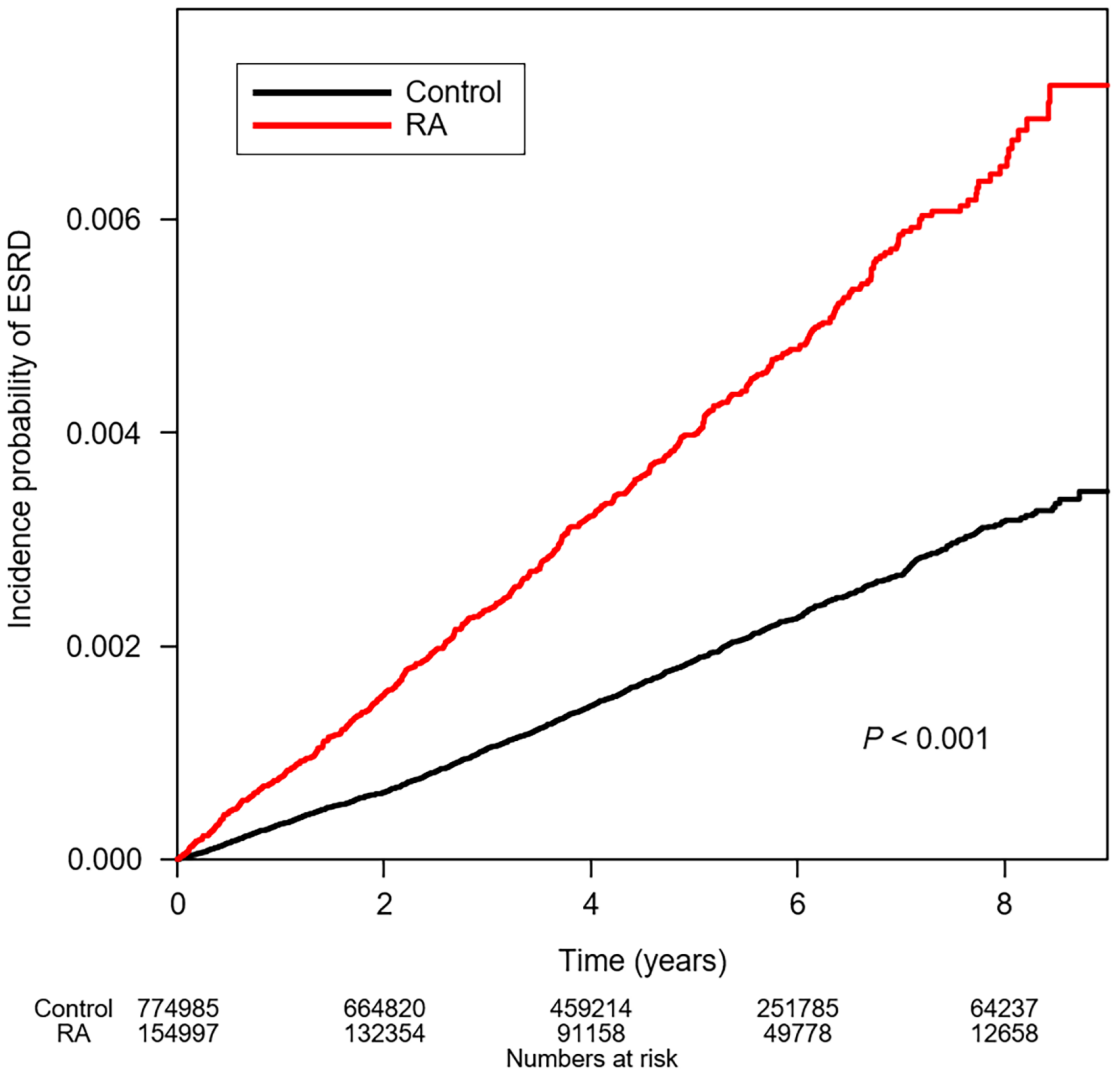
Kaplan–Meier curve for cumulative incidence of ESRD by RA. *p* value by Log-rank test. ESRD, end-stage renal disease; RA, rheumatoid arthritis.

To compare the cumulative incidences of ESRD among the subjects with or without RA, Kaplan–Meier analyses were conducted (Figure 2), which visualized that the risks of ESRD are significantly increased in the subjects with RA (*p* < 0.001, by Log-rank test). To define the independent association of RA and ESRD, Cox regression models were analyzed (Table 2). The median follow-up periods were 3,663,252.01, and 728,414.58 person-years in the subjects without RA and in the subjects with RA, respectively. During follow-up, ESRD developed in 1,370 subjects without RA and in 590 subjects with RA. Compared to the subjects without RA, the subjects with RA resulted in an increased incidence of ESRD (incidence rates of 0.374 versus 0.810 cases per 1,000 person-years). Accordingly, compared to the subjects without RA, both unadjusted (unadjusted HR 2.166, 95% CI 1.967–2.386) and fully adjusted (adjusted HR 2.153, 95% CI 1.948–2.379) models demonstrated that the risk of ESRD is significantly increased among the subjects with RA.

**Table 2:** Cox regression analysis of rheumatoid arthritis (RA) for the risk of end-stage renal disease (ESRD).

	Total number	Event number	Follow-up duration (person-years)	Incidence *per* 1,000 person-years	Model 1	Model 2	Model 3	Model 4
					HR (95%CIs)	HR (95%CIs)	HR (95%CIs)	HR (95%CIs)
Control	774,985	1,370	3,663,252.01	0.374	Reference	Reference	Reference	Reference
RA	154,997	5,90	728,414.58	0.810	2.166 (1.967, 2.386)	2.181 (1.980, 2.402)	2.160 (1.962, 2.379)	2.153 (1.948, 2.379)

Model 1, unadjusted model. Model 2, model 1 + adjusted for age and sex. Model 3, model 2 + adjusted for smoking status, alcohol consumption, regular exercise, low income, and obesity. Model 4, model 3 + adjusted for comorbid conditions, including NSAID medication, DM, HTN, DL, and CKD.

CI, confidence interval; HR, hazard ratio; RA, rheumatoid arthritis.

### 3.3. Subgroup analyses

To examine whether the association of RA with the risk of ESRD is modified by certain clinical contexts, we conducted a series of subgroup analyses (Table 3). In terms of interaction, the association of RA with the risk of ESRD was significantly more prominent in those with relatively young age (*P* for interaction < 0.001) and in those with alcohol consumption (*P* for interaction = 0.021). The association of RA with the risk of ESRD was also more strongly observed in those without comorbid conditions, such as obesity (*P* for interaction < 0.001), DM (*P* for interaction < 0.001), HTN (*P* for interaction < 0.001), DL (*P* for interaction < 0.001), and CKD (*P* for interaction < 0.001). The association of RA with the risk of ESRD was not altered by sex, smoking status, regular exercise, or low-income status.

**Table 3:** Cox regression analysis of RA for the risk of ESRD in various subgroups.

			Total number	Event number	Follow-up duration (person-years)	Incidence *per* 1,000 person-years	Model 1	Model 2	Model 3	Model 4
							HR (95%CIs)	HR (95%CIs)	HR (95%CIs)	HR (95%CIs)
Age (years)	20–39	Control	65,640	20	310168.46	0.064	Reference	Reference	Reference	Reference
		RA	13,128	33	61868.27	0.533	8.273 (4.747, 14.417)	8.273 (4.747, 14.417)	8.409 (4.822,14.666)	5.978 (3.317, 10.807)
	40–64	Control	522,990	613	2512013.56	0.244	Reference	Reference	Reference	Reference
		RA	104,598	314	500883.89	0.627	2.569 (2.243, 2.944)	2.574 (2.246, 2.949)	2.539 (2.216, 2.909)	2.617 (2.269, 3.020)
	65 ≤	Control	186,355	737	841070.00	0.876	Reference	Reference	Reference	Reference
		RA	37,271	243	165662.42	1.467	1.676 (1.450, 1.938)	1.684 (1.457, 1.947)	1.670 (1.445, 1.931)	1.645 (1.419, 1.908)
	*P* for interaction						< 0.001	< 0.001	< 0.001	< 0.001
Sex	Male	Control	207,740	572	934018.80	0.612	Reference	Reference	Reference	Reference
		RA	41,548	225	184635.16	1.219	1.991 (1.707, 2.323)	2.011 (1.723, 2.346)	1.980 (1.696, 2.310)	1.906 (1.624, 2.237)
	Female	Control	567,245	798	2729233.21	0.292	Reference	Reference	Reference	Reference
		RA	113,449	365	543779.41	0.671	2.296 (2.029, 2.599)	2.302 (2.034, 2.606)	2.289 (2.022, 2.590)	2.328 (2.049, 2.645)
	*P* for interaction						0.158	0.177	0.135	0.159
Smoking status	Former or non-smoker	Control	681,555	1,170	3232177.18	0.362	Reference	Reference	Reference	Reference
		RA	136,803	497	645888.10	0.769	2.126 (1.914, 2.362)	2.136 (1.923, 2.372)	2.112 (1.902, 2.346)	2.126 (1.908, 2.370)
	Current smoker	Control	93,430	200	431074.83	0.464	Reference	Reference	Reference	Reference
		RA	18,194	93	82526.47	1.127	2.431 (1.901, 3.109)	2.456 (1.920, 3.141)	2.445 (1.911, 3.129)	2.322 (1.795, 3.005)
	*P* for interaction						0.325	0.288	0.326	0.344
Alcohol consumption	No	Control	526,663	1,068	2519363.34	0.424	Reference	Reference	Reference	Reference
		RA	109,173	444	518451.40	0.856	2.021 (1.809, 2.257)	2.028 (1.816, 2.266)	2.029 (1.817, 2.267)	2.057 (1.835, 2.306)
	Yes	Control	248,322	302	1143888.68	0.264	Reference	Reference	Reference	Reference
		RA	45,824	146	209963.17	0.695	2.636 (2.163, 3.211)	2.658 (2.181, 3.238)	2.666 (2.188, 3.249)	2.477 (2.016, 3.043)
	*P* for interaction						0.022	0.020	0.021	0.021
Regular exercise	No	Control	620,803	1,144	2945086.91	0.388	Reference	Reference	Reference	Reference
		RA	126,370	490	595652.33	0.823	2.118 (1.906, 2.355)	2.130 (1.916, 2.368)	2.121 (1.908, 2.357)	2.057 (1.835, 2.306)
	Yes	Control	154,182	226	718165.11	0.315	Reference	Reference	Reference	Reference
		RA	28,627	100	132762.25	0.753	2.395 (1.893, 3.031)	2.414 (1.908, 3.055)	2.379 (1.879, 3.010)	2.477 (2.016, 3.043)
	*P* for interaction						0.353	0.351	0.374	0.330
Low-income status	No	Control	615,853	1,043	2911146.40	0.358	Reference	Reference	Reference	Reference
		RA	123,688	454	582702.24	0.779	2.175 (1.948, 2.428)	2.179 (1.951, 2.433)	2.154 (1.929, 2.405)	2.146 (1.915, 2.405)
	Yes	Control	159,132	327	752105.61	0.435	Reference	Reference	Reference	Reference
		RA	31,309	136	145712.33	0.933	2.144 (1.756, 2.619)	2.203 (1.804, 2.691)	2.178 (1.783, 2.661)	2.183 (1.775, 2.685)
	*P* for interaction						0.922	0.893	0.858	0.767
Obesity	No	Control	514,052	794	2436900.49	0.326	Reference	Reference	Reference	Reference
		RA	103,344	387	487430.47	0.794	2.437 (2.158, 2.752)	2.466 (2.184, 2.785)	2.441 (2.161, 2.756)	2.463 (2.171, 2.794)
	Yes	Control	260,933	576	1226351.52	0.470	Reference	Reference	Reference	Reference
		RA	51,653	203	240984.11	0.842	1.795 (1.530, 2.106)	1.789 (1.525, 2.100)	1.775 (1.512, 2.083)	1.742 (1.478, 2.053)
	*P* for interaction						0.003	0.002	0.002	< 0.001
DM	No	Control	680,155	622	3237323.27	0.192	Reference	Reference	Reference	Reference
		RA	135,526	402	642408.63	0.626	3.257 (2.873, 3.693)	3.276 (2.890, 3.714)	3.253 (2.869, 3.688)	3.214 (2.819, 3.664)
	Yes	Control	94,830	748	425928.74	1.756	Reference	Reference	Reference	Reference
		RA	19,471	188	86005.94	2.186	1.247 (1.063, 1.463)	1.258 (1.072, 1.476)	1.238 (1.055, 1.453)	1.273 (1.081, 1.500)
	*P* for interaction						< 0.001	< 0.001	< 0.001	< 0.001
HTN	No	Control	499,164	178	2366300.13	0.075	Reference	Reference	Reference	Reference
		RA	96,288	182	455905.25	0.399	5.308 (4.317, 6.526)	5.343 (4.346, 6.570)	5.315 (4.322, 6.536)	5.760 (4.609, 7.200)
	Yes	Control	275,821	1,192	1296951.88	0.919	Reference	Reference	Reference	Reference
		RA	58,709	408	272509.33	1.497	1.630 (1.457, 1.824)	1.659 (1.483, 1.856)	1.635 (1.461, 1.829)	1.672 (1.489, 1.877)
	*P* for interaction						< 0.001	< 0.001	< 0.001	< 0.001
DL	No	Control	533,388	544	2573225.43	0.211	Reference	Reference	Reference	Reference
		RA	102,586	330	493340.84	0.669	3.165 (2.760, 3.628)	3.224 (2.812, 3.697)	3.196 (2.787, 3.665)	3.240 (2.808, 3.740)
	Yes	Control	241,597	826	1090026.59	0.758	Reference	Reference	Reference	Reference
		RA	52,411	260	235073.73	1.106	1.460 (1.270, 1.679)	1.463 (1.273, 1.682)	1.448 (1.260, 1.665)	1.506 (1.305, 1.737)
	*P* for interaction						< 0.001	< 0.001	< 0.001	< 0.001
CKD	No	Control	730,211	461	3443455.12	0.134	Reference	Reference	Reference	Reference
		RA	145,873	345	684097.35	0.504	3.770 (3.279, 4.335)	3.789 (3.296, 4.356)	3.767 (3.276, 4.331)	3.635 (3.143, 4.204)
	Yes	Control	44,774	909	219796.89	4.136	Reference	Reference	Reference	Reference
		RA	9,124	245	44317.23	5.528	1.337 (1.161, 1.539)	1.340 (1.164, 1.543)	1.332 (1.156, 1.533)	1.357 (1.175, 1.569)
	*P* for interaction						< 0.001	< 0.001	< 0.001	< 0.001

Model 1, unadjusted model. Model 2, model 1 + adjusted for age and sex. Model 3, model 2 + adjusted for smoking status, alcohol consumption, regular exercise, low income, and obesity. Model 4, model 3 + adjusted for comorbid conditions, including NSAID medication, DM, HTN, DL, and CKD.

CI, confidence interval; CKD, chronic kidney disease; DL, dyslipidemia; DM, diabetes mellitus; HR, hazard ratio; HTN, hypertension; RA, rheumatoid arthritis.

### 3.4. Sensitivity analyses

To evaluate the possibility of reverse causation, a sensitivity analysis was performed by excluding the subjects with ESRD occurring within 3 years (Supplementary Table 1) and 5 years (Supplementary Table 2) of follow-up. Both models excluding the subjects with ESRD occurring within 3 years (adjusted HR 1.989, 95% CI 1.746–2.256) and 5 years (adjusted HR 1.944, 95% CI 1.623–2.328) demonstrated a significant association between RA and the risk of ESRD, suggesting the least likelihood of reverse causation. Next, we additionally used cause-specific hazard models, in which the death occurring before reaching the primary outcome was treated as a competing risk and censored (Supplementary Table 3). Even in this competing risk analysis model, RA was significantly associated with the risk of ESRD (adjusted HR 2.121, 95% CI 1.917–2.347), supporting the robustness of the association. Finally, the propensity score-matching analysis was adopted to balance the differences in covariates, which demonstrated much improved absolute standardized mean difference, especially for the use of NSAIDs, compared to that before propensity score-matching (Supplementary Table 4). The risk of ESRD associated with RA was still significant both in the model before (adjusted HR 2.142, 95% CI 1.857–2.470) and after (adjusted HR 2.130, 95% CI 1.847–2.457) adoption of competing risk analysis (Supplementary Table 5).

## 4. Discussion

In the present study, we found that RA is associated with an increased risk of ESRD, and that the association is significantly altered by various clinical contexts, such as age and comorbid conditions. Our finding is robust, because we demonstrated consistent results in a series of sensitivity analyses by excluding the subjects with ESRD occurring within 3 and 5 years of follow-up, and by adopting cause-specific hazard models, in which the death occurring before reaching the primary outcome was treated as a competing risk and censored. In addition, despite the substantial differences in the baseline characteristics, the prevalence of CKD among the subjects with or without RA was similar at the baseline. Moreover, eGFR was slightly, but significantly higher in the subjects with RA at the baseline. These all collectively suggest that RA increased the risk of ESRD in the affected individuals during the course of the disease.

It is of note that, contrary to the previous reports (17, 18), RA is not only associated with the development of CKD, but also associated with the progression of CKD to ESRD. This suggests that clinicians managing the patients with RA should also consider any strategies to protect the kidney concurrently, as RA usually requires life-long therapy. In this regard, the result from the subgroup analyses in the current study presents a valuable insight, in that the risk of ESRD among the patients with RA is significantly higher in the subjects with relatively young age. We assume that the risk of ESRD imposed by RA accumulates, and that the young patients have temporally more chance to be exposed to the cumulative risk of ESRD during the course of RA.

The precise mechanisms to associate RA and ERSD are not clearly demonstrated in the present study, while some possibilities could be speculated. Although a relatively higher prevalence of comorbid conditions, such as DM and HTN, which are well-known risk factors of CKD progression, has been reported among the patients with RA (11), the result from the subgroup analyses in the current study suggests that RA is directly, rather than the aggravation of underlying comorbidities, associated with the progression of CKD, as the association of RA with the risk of ESRD was significantly stronger among those without obesity, DM, HTN, DL, or CKD. It is assumed that the relative risk of ESRD attributable to RA may be attenuated if the subjects are already affected by the traditional risk factors for ESRD, such as obesity, DM, HTN, DL, and CKD. Indeed, RA is associated with chronic inflammation in vascular beds (9, 10), and may potentially increase the risk of development and progression of CKD due to the accelerated atherosclerosis. In addition, medications such as some DMARDs or NSAIDs may directly affect the kidney function (14–16), though the potential contribution of herbal and over-the-counter medications to the progression of CKD should also be considered. As the present study did not analyze individual medications used in the subjects, further studies to unveil the contribution of RA medication to the risk of ESRD should be warranted.

Regardless of the precise mechanism of the association between RA and ESRD, it seems prudent that RA patients who are relatively young and healthy (i.e., without other comorbid conditions) should be prioritized to be managed by a kidney-protective treatment strategy, such as biologic agents. A guideline already recommends to control disease activity to lower cardiovascular risk in patients with RA (28), as meta-analyses identified that the use of tumor necrosis factor inhibitors is beneficial to reduce the risk of cardiovascular events in patients with RA (29, 30). Likewise, a recent study reported that the use of biologic agents lowers the risk of incident CKD (31), whereas the use of biologics including etanercept, adalimumab, infliximab, abatacept, certolizumab, golimumab, rituximab, tocilizumab, and anakinra was associated with lower risk of incident CKD. The authors suggested that improved pain management resulting from the use of biologic treatment may help to reduce the need for potentially nephrotoxic anti-inflammatory agents such as NSAIDs and certain types of non-biologic DMARDs like D-penicillamine and cyclosporine. Based on the findings of the present study that the risk of ESRD imposed by RA is relatively higher in relatively young and healthy individuals, administration of biologic agents in this specific population should be preferentially considered among these patients with RA. In addition, protective and prophylactic measures, such as administration of nephroprotective agents including safe antioxidants and anti-inflammatory agents as supplementation, to decrease the incidence of ESRD among RA patients should also be established. For instance, omega-3 fatty acid attenuated methotrexate-induced nephrotoxicity in a pre-clinical trial (32), although it is necessary to further validate whether the use of omega-3 fatty acid lowers the risk of ESRD in patients with RA. In this context, it is worthy of note that Hagar et al. reported that the introduction of pharmaceutical care services in RA patient treatment protocol effectively resulted in an improvement in the detection and prevention of drug-related problems and showed a significant reduction in Disease Activity Score 28, Health Assessment Questionnaire, and Rheumatoid Arthritis Quality of Life Questionnaire scores (33).

Recently, it has been reported that oral administration of 1 g/day of vitamin C for a period of 16 weeks resulted in the reduction of serum levels of malondialdehyde and elevation of glutathione peroxidase levels among the patients on maintenance hemodialysis (34). Additionally, supplementation with omega-3 fatty in patients with ESRD significantly reduced total cholesterol level and oxidative stress markers (35). Another study was carried out to evaluate the effect of oral febuxostat on the endothelial dysfunction in patients with ESRD, which lead to the improvement in hyperuricemia and endothelial dysfunction by febuxostat with no safety concerns (36). Therefore, it is also required to evaluate whether the use of vitamin C, omega-3 fatty acid, or febuxostat could lower the risk of ESRD in patients with RA.

There are several limitations to be acknowledged in the current study. First, due to the retrospective nature of the study, a causal association between RA and ESRD could not be confirmed. Second, we did not analyze the effect of individual medications, except the use of NSAIDs, on the renal prognosis among the patients with RA. Third, similarly, we considered only limited medical conditions that may affect the kidney outcomes in the subjects with or without RA. Fourth, we are not able to suggest a precise mechanism for the increased risk of ESRD among the patients with RA, because the current study is not interventional. Fifth, an extrapolation of the data to other populations requires precaution, as the study population in this study is from a single country.

In conclusion, we report that RA is associated with increased risk of ESRD. Our finding is robust, because we demonstrated consistent results in a series of sensitivity analyses. Moreover, eGFR was slightly, but significantly higher in the subjects with RA at the baseline. As the risk of ESRD imposed by RA is relatively higher in relatively young individuals not suffering from any other medical conditions, such as obesity, DM, HTN, DL, and CKD, kidney-protective treatment, such as biologic agents, should be preferentially considered among these patients with RA.

## Data availability statement

The raw data supporting the conclusions of this article will be made available by the authors, without undue reservation.

## Ethics statement

The institutional review board of Chonnam National University Hospital approved the study protocol (CNUH-EXP-2021-431). Patient identification numbers were anonymized to protect individual privacy. As all data to identify the individual patients were anonymized and deidentified for analysis, the institutional review board waived the need for informed consent. Written informed consent for participation was not required for this study in accordance with the national legislation and the institutional requirements.

## Author contributions

SS and JJ conceived, designed, and performed the analysis. SS interpreted the results and drafted the manuscript. JJ, TO, EY, HC, CK, EB, SM, K-DH, and SK provided revisions to the manuscript. All authors contributed to the article and approved the submitted version.

## Funding

This work was supported by a grant of Chonnam National University Hospital Biomedical Research Institute (BCRI22079, BCRI22042, and BCRI22046), and by the National Research Foundation of Korea (NRF) funded by the Korea Government (MSIT; NRF-2019R1A2C2086276, NRF-2020R1F1A1074001).

## Conflict of interest

The authors declare that the research was conducted in the absence of any commercial or financial relationships that could be construed as a potential conflict of interest.

## Publisher’s note

All claims expressed in this article are solely those of the authors and do not necessarily represent those of their affiliated organizations, or those of the publisher, the editors and the reviewers. Any product that may be evaluated in this article, or claim that may be made by its manufacturer, is not guaranteed or endorsed by the publisher.

## Footnotes

^1^ https://nhiss.nhis.or.kr/bd/ab/bdaba000eng.do
