# Supplementary material for: Rheumatoid arthritis and the risk of end-stage renal disease: A nationwide, population-based study
Source: Front Med (Lausanne). 2023 Feb 2;10:1116489. doi: 10.3389/fmed.2023.1116489 (PMC9932810; doi:10.3389/fmed.2023.1116489)
Supplement: Supplementary file 1 [file Table_1.DOCX]

**- Supplementary Materials -**

**Rheumatoid arthritis and the risk of end-stage renal disease: a nation-wide, population-based study**

Sang Heon Suh^1^, Jin Hyung Jung^2^, Tae Ryom Oh^1^, Eun Mi Yang^3^, Hong Sang Choi^1^, Chang Seong Kim^1^, Eun Hui Bae^1^, Seong Kwon Ma^1^, Kyung-Do Han4^4*^, Soo Wan Kim^1*^

^1^ Department of Internal Medicine, Chonnam National University Medical School and Chonnam National University Hospital, Gwangju 61469, Korea

^2^ Department of Biostatistics, College of Medicine, Catholic University of Korea, Seoul 03083, Korea

^3^ Department of Pediatrics, Chonnam National University Medical School and Chonnam National University Hospital, Gwangju 61469, Korea

^4^ Department of Statistics and Actuarial Science, Soongsil University, Seoul 06978, Korea

**Corresponding authors**

* Kyung-Do Han, Ph.D., Department of Statistics and Actuarial Science, Soongsil University, Seoul 06978, Korea, Tel: +82-2-820-7025, Fax: +82-2-823-1746, Email: hkd917@naver.com

*Soo Wan Kim, M.D., Ph.D., Department of Internal Medicine, Chonnam National University Medical School, 42 Jebongro, Gwangju 61469, Korea, Tel: +82-62-225-6271, Fax: +82-62-225-8578, Email: skimw@chonnam.ac.kr

**Table of contents**

Supplementary Table 1. Cox regression analysis of RA for the risk of ESRD after excluding the subjects with ESRD occurring within 3 years of follow-up

Supplementary Table 2. Cox regression analysis of RA for the risk of ESRD after excluding the subjects with ESRD occurring within 5 years of follow-up

Supplementary Table 3. Cause-specific Cox regression analysis of RA for the risk of ESRD

Supplementary Table 4. Propensity score matching analysis for the RA group

Supplementary Table 5. Cox regression analysis of RA for the risk of ESRD after propensity score matching

**Supplementary Table 1. Cox regression analysis of RA for the risk of ESRD after excluding the subjects with ESRD occurring within 3 years of follow-up**

|  | |  | Total number | Event number | Follow-up duration (person-years) | Incidence *per* 1,000  person-years | Model 1 | Model 2 | Model 3 | Model 4 |
| --- | --- | --- | --- | --- | --- | --- | --- | --- | --- | --- |
|  |  |  |  |  |  |  | HR  (95%CIs) | HR  (95%CIs) | HR  (95%CIs) | HR  (95%CIs) |
| Total | | Control | 664820 | 899 | 2172612.6 | 0.414 | Reference | Reference | Reference | Reference |
|  |  | RA | 132354 | 361 | 431006.9 | 0.838 | 2.024  (1.792, 2.287) | 2.043  (1.808, 2.308) | 2.027  (1.794, 2.29) | 1.989  (1.746, 2.256) |
| Age (years) | 20 – 39 | Control | 56642 | 13 | 183541.1 | 0.071 | Reference | Reference | Reference | Reference |
|  |  | RA | 11301 | 20 | 36579.9 | 0.547 | 7.721  (3.841, 15.521) | 7.72  (3.840, 15.519) | 7.827  (3.889, 15.755) | 5.256  (2.543, 10.863) |
|  | 40 – 64 | Control | 452663 | 407 | 1502967.9 | 0.271 | Reference | Reference | Reference | Reference |
|  |  | RA | 90318 | 188 | 299314.5 | 0.628 | 2.319  (1.951, 2.757) | 2.324  (1.955, 2.763) | 2.301  (1.935, 2.735) | 2.345  (1.957, 2.810) |
|  | 65 ≤ | Control | 155515 | 479 | 486103.7 | 0.985 | Reference | Reference | Reference | Reference |
|  |  | RA | 30735 | 153 | 95112.5 | 1.609 | 1.634  (1.362, 1.960) | 1.644  (1.371, 1.973) | 1.63  (1.359, 1.956) | 1.581  (1.313, 1.902) |
|  | *P* for interaction |  |  |  |  |  | < 0.001 | < 0.001 | < 0.001 | < 0.001 |
| Sex | Male | Control | 173147 | 375 | 537798.5 | 0.697 | Reference | Reference | Reference | Reference |
|  |  | RA | 34288 | 133 | 105816.9 | 1.257 | 1.803  (1.479, 2.197) | 1.827  (1.499, 2.226) | 1.809  (1.484, 2.205) | 1.725  (1.405, 2.117) |
|  | Female | Control | 491673 | 524 | 1634814.1 | 0.321 | Reference | Reference | Reference | Reference |
|  |  | RA | 98066 | 228 | 325190.0 | 0.701 | 2.188  (1.873, 2.556) | 2.196  (1.880, 2.565) | 2.184  (1.869, 2.552) | 2.176  (1.855, 2.552) |
|  | *P* for interaction |  |  |  |  |  | 0.132 | 0.150 | 0.122 | 0.098 |
| Smoking status | Former or non-smoker | Control | 585749 | 771 | 1920477.2 | 0.401 | Reference | Reference | Reference | Reference |
|  |  | RA | 117162 | 304 | 383119.4 | 0.793 | 1.977  (1.731, 2.257) | 1.989  (1.742, 2.272) | 1.970  (1.725, 2.250) | 1.961  (1.712, 2.249) |
|  | Current smoker | Control | 79071 | 128 | 252135.4 | 0.508 | Reference | Reference | Reference | Reference |
|  |  | RA | 15192 | 57 | 47887.5 | 1.190 | 2.344  (1.716, 3.203) | 2.388  (1.747, 3.263) | 2.39  (1.748, 3.267) | 2.174  (1.571, 3.008) |
|  | *P* for interaction |  |  |  |  |  | 0.323 | 0.289 | 0.311 | 0.384 |
| Alcohol consumption | No | Control | 454483 | 692 | 1505205.3 | 0.460 | Reference | Reference | Reference | Reference |
|  |  | RA | 93669 | 264 | 308761.5 | 0.855 | 1.860  (1.614, 2.143) | 1.871  (1.624, 2.156) | 1.871  (1.623, 2.156) | 1.865  (1.612, 2.158) |
|  | Yes | Control | 210337 | 207 | 667407.3 | 0.310 | Reference | Reference | Reference | Reference |
|  |  | RA | 38685 | 97 | 122245.4 | 0.793 | 2.559  (2.011, 3.257) | 2.59  (2.035, 3.296) | 2.598  (2.041, 3.307) | 2.402  (1.870, 3.085) |
|  | *P* for interaction |  |  |  |  |  | 0.025 | 0.023 | 0.024 | 0.025 |
| Regular exercise | No | Control | 533625 | 746 | 1750502.7 | 0.426 | Reference | Reference | Reference | Reference |
|  |  | RA | 108102 | 296 | 353091.3 | 0.838 | 1.967  (1.719, 2.251) | 1.982  (1.732, 2.268) | 1.975  (1.726, 2.260) | 1.940  (1.689, 2.229) |
|  | Yes | Control | 131195 | 153 | 422109.9 | 0.362 | Reference | Reference | Reference | Reference |
|  |  | RA | 24252 | 65 | 77915.6 | 0.834 | 2.303  (1.723, 3.079) | 2.332  (1.745, 3.117) | 2.299  (1.719, 3.073) | 2.237  (1.657, 3.018) |
|  | *P* for interaction |  |  |  |  |  | 0.336 | 0.329 | 0.346 | 0.334 |
| Low-income status | No | Control | 528245 | 700 | 1726486.9 | 0.405 | Reference | Reference | Reference | Reference |
|  |  | RA | 105718 | 281 | 345311.3 | 0.814 | 2.007  (1.748, 2.305) | 2.016  (1.755, 2.315) | 1.998  (1.740, 2.295) | 1.965  (1.704, 2.267) |
|  | Yes | Control | 136575 | 199 | 446125.7 | 0.446 | Reference | Reference | Reference | Reference |
|  |  | RA | 26636 | 80 | 85695.6 | 0.934 | 2.088  (1.611, 2.707) | 2.148  (1.657, 2.784) | 2.124  (1.639, 2.754) | 2.083  (1.594, 2.724) |
|  | *P* for interaction |  |  |  |  |  | 0.779 | 0.643 | 0.626 | 0.557 |
| Obesity | No | Control | 442457 | 509 | 1447509.7 | 0.352 | Reference | Reference | Reference | Reference |
|  |  | RA | 88589 | 233 | 288999.9 | 0.806 | 2.293  (1.964, 2.678) | 2.327  (1.993, 2.718) | 2.304  (1.973, 2.691) | 2.255  (1.921, 2.647) |
|  | Yes | Control | 222363 | 390 | 725102.9 | 0.538 | Reference | Reference | Reference | Reference |
|  |  | RA | 43765 | 128 | 142007.0 | 0.901 | 1.676  (1.373, 2.047) | 1.673  (1.370, 2.043) | 1.665  (1.364, 2.033) | 1.217  (1.339, 2.017) |
|  | *P* for interaction |  |  |  |  |  | 0.015 | 0.010 | 0.011 | 0.008 |
| DM | No | Control | 586087 | 415 | 1927047.1 | 0.215 | Reference | Reference | Reference | Reference |
|  |  | RA | 116286 | 243 | 381901.7 | 0.636 | 2.955  (2.522, 3.462) | 2.976  (2.540, 3.486) | 2.960  (2.526, 3.468) | 2.893  (2.453, 3.411) |
|  | Yes | Control | 78733 | 484 | 245565.5 | 1.971 | Reference | Reference | Reference | Reference |
|  |  | RA | 16068 | 118 | 49105.2 | 2.403 | 1.220  (0.997, 1.492) | 1.233  (1.008, 1.508) | 1.218  (0.996, 1.490) | 1.217  (0.991, 1.494) |
|  | *P* for interaction |  |  |  |  |  | < 0.001 | < 0.001 | < 0.001 | < 0.001 |
| HTN | No | Control | 429443 | 128 | 1404642.8 | 0.091 | Reference | Reference | Reference | Reference |
|  |  | RA | 82702 | 123 | 270631.3 | 0.454 | 4.988  (3.895, 6.389) | 5.021  (3.920, 6.431) | 4.991  (3.896, 6.394) | 5.228  (4.011, 6.813) |
|  | Yes | Control | 235377 | 771 | 767969.8 | 1.004 | Reference | Reference | Reference | Reference |
|  |  | RA | 49652 | 238 | 160375.6 | 1.484 | 1.479  (1.279, 1.711) | 1.508  (1.304, 1.744) | 1.491  (1.290, 1.725) | 1.500  (1.293, 1.741) |
|  | *P* for interaction |  |  |  |  |  | < 0.001 | < 0.001 | < 0.001 | < 0.001 |
| DL | No | Control | 463114 | 385 | 1544145.3 | 0.249 | Reference | Reference | Reference | Reference |
|  |  | RA | 88815 | 216 | 295758.2 | 0.730 | 2.929  (2.480, 3.460) | 2.993  (2.533, 3.536) | 2.965  (2.510, 3.503) | 3.004  (2.525, 3.576) |
|  | Yes | Control | 201706 | 514 | 628467.3 | 0.818 | Reference | Reference | Reference | Reference |
|  |  | RA | 43539 | 145 | 135248.7 | 1.072 | 1.311  (1.090, 1.576) | 1.313  (1.092, 1.579) | 1.305  (1.086, 1.570) | 1.314  (1.089, 1.586) |
|  | *P* for interaction |  |  |  |  |  | < 0.001 | < 0.001 | < 0.001 | < 0.001 |
| CKD | No | Control | 626362 | 344 | 2038410.5 | 0.169 | Reference | Reference | Reference | Reference |
|  |  | RA | 124633 | 215 | 403996.9 | 0.532 | 3.155  (2.660, 3.741) | 3.176  (2.678, 3.766) | 3.159  (2.663, 3.746) | 3.028  (2.538, 3.614) |
|  | Yes | Control | 38458 | 555 | 134202.1 | 4.136 | Reference | Reference | Reference | Reference |
|  |  | RA | 7721 | 146 | 27010.0 | 5.405 | 1.307  (1.089, 1.568) | 1.312  (1.094, 1.575) | 1.305  (1.088, 1.566) | 1.311  (1.088, 1.580) |
|  | *P* for interaction |  |  |  |  |  | < 0.001 | < 0.001 | < 0.001 | < 0.001 |

Note: Model 1, unadjusted model. Model 2, model 1 + adjusted for age and sex. Model 3, model 2 + adjusted for smoking status, alcohol consumption, regular exercise, low income, and obesity. Model 4, model 3 + adjusted for comorbid conditions, including NSAID medication, DM, HTN, DL, and CKD. Abbreviations: CI, confidence interval; CKD, chronic kidney disease; DL, dyslipidemia; DM, diabetes mellitus; HR, hazard ratio; HTN, hypertension; RA, rheumatoid arthritis.

**Supplementary Table 2. Cox regression analysis of RA for the risk of ESRD after excluding the subjects with ESRD occurring within 5 years of follow-up**

|  | |  | Total number | Event number | Follow-up duration (person-years) | Incidence *per* 1,000  person-years | Model 1 | Model 2 | Model 3 | Model 4 |
| --- | --- | --- | --- | --- | --- | --- | --- | --- | --- | --- |
|  |  |  |  |  |  |  | HR  (95%CIs) | HR  (95%CIs) | HR  (95%CIs) | HR  (95%CIs) |
| Total | | Control | 459214 | 447 | 1048924.6 | 0.426 | Reference | Reference | Reference | Reference |
|  |  | RA | 91158 | 174 | 207588.6 | 0.838 | 1.967  (1.651, 2.343) | 1.991  (1.671, 2.372) | 1.980  (1.661, 2.359) | 1.944  (1.623, 2.328) |
| Age (years) | 20 – 39 | Control | 39051 | 5 | 87831.1 | 0.057 | Reference | Reference | Reference | Reference |
|  |  | RA | 7786 | 13 | 17487.6 | 0.743 | 13.06  (4.656, 36.632) | 13.062  (4.657, 36.639) | 13.484  (4.799, 37.89) | 10.353  (3.494, 30.675) |
|  | 40 – 64 | Control | 317694 | 203 | 732401.4 | 0.277 | Reference | Reference | Reference | Reference |
|  |  | RA | 63277 | 85 | 145665.5 | 0.584 | 2.105  (1.634, 2.712) | 2.112  (1.640, 2.721) | 2.097  (1.627, 2.701) | 2.146  (1.648, 2.794) |
|  | 65 ≤ | Control | 102469 | 239 | 228692.1 | 1.045 | Reference | Reference | Reference | Reference |
|  |  | RA | 20095 | 76 | 44435.6 | 1.710 | 1.637  (1.265, 2.119) | 1.648  (1.273, 2.133) | 1.636  (1.264, 2.118) | 1.601  (1.231, 2.081) |
|  | *P* for interaction |  |  |  |  |  | 0.001 | 0.001 | 0.001 | 0.001 |
| Sex | Male | Control | 113913 | 181 | 250565.3 | 0.722 | Reference | Reference | Reference | Reference |
|  |  | RA | 22447 | 62 | 49082.5 | 1.263 | 1.748  (1.310, 2.333) | 1.776  (1.331, 2.370) | 1.754  (1.314, 2.342) | 1.688  (1.251, 2.081) |
|  | Female | Control | 345301 | 266 | 798359.3 | 0.333 | Reference | Reference | Reference | Reference |
|  |  | RA | 68711 | 112 | 158506.1 | 0.707 | 2.121.  (1.701, 2.645) | 2.134  (1.711, 2.661) | 2.127  (1.706, 2.653) | 2.114  (1.687, 2.650) |
|  | *P* for interaction |  |  |  |  |  | 0.298 | 0.328 | 0.298 | 0.228 |
| Smoking status | Former or non-smoker | Control | 405559 | 387 | 929879.8 | 0.416 | Reference | Reference | Reference | Reference |
|  |  | RA | 80926 | 147 | 185163.7 | 0.794 | 1.908  (1.578, 2.306) | 1.925  (1.592, 2.328) | 1.912  (1.581, 2.312) | 1.907  (1.569, 2.318) |
|  | Current smoker | Control | 53655 | 60 | 119044.8 | 0.504 | Reference | Reference | Reference | Reference |
|  |  | RA | 10232 | 27 | 22425.0 | 1.204 | 2.387  (1.516, 3.760) | 2.436  (1.546, 3.836) | 2.424  (1.538, 3.821) | 2.181  (1.358, 3.501) |
|  | *P* for interaction |  |  |  |  |  | 0.370 | 0.331 | 0.345 | 0.417 |
| Alcohol consumption | No | Control | 317805 | 345 | 733346.3 | 0.470 | Reference | Reference | Reference | Reference |
|  |  | RA | 65210 | 121 | 150015.7 | 0.807 | 1.715  (1.394, 2.109) | 1.731  (1.407, 2.129) | 1.728  (1.405, 2.126) | 1.722  (1.393, 2.130) |
|  | Yes | Control | 141409 | 102 | 315578.3 | 0.323 | Reference | Reference | Reference | Reference |
|  |  | RA | 25948 | 53 | 57572.9 | 0.921 | 2.848  (2.044, 3.969) | 2.895  (2.077, 4.035) | 2.910  (2.088, 4.057) | 2.732  (1.934, 3.860) |
|  | *P* for interaction |  |  |  |  |  | 0.011 | 0.010 | 0.010 | 0.010 |
| Regular exercise | No | Control | 369930 | 366 | 847302.1 | 0.432 | Reference | Reference | Reference | Reference |
|  |  | RA | 74622 | 138 | 170499.2 | 0.809 | 1.874  (1.541, 2.279) | 1.895  (1.558, 2.305) | 1.888  (1.552, 2.296) | 1.864  (1.524, 2.279) |
|  | Yes | Control | 89284 | 81 | 201622.5 | 0.402 | Reference | Reference | Reference | Reference |
|  |  | RA | 16536 | 36 | 37089.4 | 0.971 | 2.418  (1.633, 3.581) | 2.450  (1.654, 3.628) | 2.434  (1.644, 3.606) | 2.349  (1.566, 3.523) |
|  | *P* for interaction |  |  |  |  |  | 0.256 | 0.249 | 0.258 | 0.296 |
| Low-income status | No | Control | 364717 | 359 | 834077.3 | 0.430 | Reference | Reference | Reference | Reference |
|  |  | RA | 72941 | 139 | 166725.9 | 0.834 | 1.937  (1.592, 2.356) | 1.951  (1.604, 2.373) | 1.940  (1.595, 2.360) | 1.883  (1.540, 2.302) |
|  | Yes | Control | 94497 | 88 | 214847.3 | 0.410 | Reference | Reference | Reference | Reference |
|  |  | RA | 18217 | 35 | 40862.8 | 0.857 | 2.085  (1.409, 3.085) | 2.157  (1.458, 3.192) | 2.140  (1.446, 3.166) | 2.226  (1.480, 3.349) |
|  | *P* for interaction |  |  |  |  |  | 0.732 | 0.646 | 0.640 | 0.655 |
| Obesity | No | Control | 306397 | 253 | 698569.0 | 0.362 | Reference | Reference | Reference | Reference |
|  |  | RA | 61224 | 100 | 139213.7 | 0.718 | 1.984  (1.574, 2.501) | 2.019  (1.602, 2.546) | 1.995  (1.583, 2.516) | 1.998  (1.573, 2.538) |
|  | Yes | Control | 152817 | 194 | 350355.6 | 0.554 | Reference | Reference | Reference | Reference |
|  |  | RA | 29934 | 74 | 68374.9 | 1.082 | 1.955  (1.496, 2.555) | 1.956  (1.497, 2.557) | 1.958  (1.498, 2.560) | 1.876  (1.426, 2.468) |
|  | *P* for interaction |  |  |  |  |  | 0.939 | 0.865 | 0.876 | 0.833 |
| DM | No | Control | 406968 | 210 | 933855.4 | 0.225 | Reference | Reference | Reference | Reference |
|  |  | RA | 80708 | 116 | 184914.4 | 0.627 | 2.790  (2.224, 3.500) | 2.816  (2.245, 3.533) | 2.814  (2.242, 3.530) | 2.701  (2.136, 3.416) |
|  | Yes | Control | 52246 | 237 | 115069.2 | 2.060 | Reference | Reference | Reference | Reference |
|  |  | RA | 10450 | 58 | 22674.2 | 2.558 | 1.242  (0.932, 1.655) | 1.257  (0.943, 1.675) | 1.238  (0.929, 1.650) | 1.249  (0.932, 1.675) |
|  | *P* for interaction |  |  |  |  |  | < 0.001 | < 0.001 | < 0.001 | < 0.001 |
| HTN | No | Control | 296808 | 71 | 678447.5 | 0.105 | Reference | Reference | Reference | Reference |
|  |  | RA | 57178 | 58 | 130793.8 | 0.443 | 4.237  (2.995, 5.994) | 4.275  (3.022, 6.047) | 4.261  (3.011, 6.029) | 4.846  (3.328, 7.058) |
|  | Yes | Control | 162406 | 376 | 370477.1 | 1.015 | Reference | Reference | Reference | Reference |
|  |  | RA | 33980 | 116 | 76794.8 | 1.511 | 1.489  (1.210, 1.834) | 1.522  (1.236, 1.874) | 1.509  (1.225, 1.858) | 1.491  (1.249, 1.844) |
|  | *P* for interaction |  |  |  |  |  | < 0.001 | < 0.001 | < 0.001 | < 0.001 |
| DL | No | Control | 326034 | 211 | 753997.2 | 0.280 | Reference | Reference | Reference | Reference |
|  |  | RA | 62426 | 103 | 144288.6 | 0.714 | 2.551  (2.015, 3.228) | 2.618  (2.068, 3.314) | 2.600  (2.054, 3.291) | 2.667  (2.086, 3.409) |
|  | Yes | Control | 133180 | 236 | 294927.4 | 0.800 | Reference | Reference | Reference | Reference |
|  |  | RA | 28732 | 71 | 63300.0 | 1.122 | 1.402  (1.075, 1.827) | 1.404  (1.077, 1.831) | 1.400  (1.073, 1.825) | 1.388  (1.059, 1.820) |
|  | *P* for interaction |  |  |  |  |  | 0.001 | 0.001 | 0.001 | 0.001 |
| CKD | No | Control | 431448 | 193 | 980941.2 | 0.197 | Reference | Reference | Reference | Reference |
|  |  | RA | 85525 | 101 | 193916.0 | 0.521 | 2.648  (2.081, 3.368) | 2.672  (2.100, 3.399) | 2.668  (2.097, 3.395) | 2.545  (1.985, 3.263) |
|  | Yes | Control | 27766 | 254 | 67983.4 | 3.736 | Reference | Reference | Reference | Reference |
|  |  | RA | 5633 | 73 | 13672.6 | 5.339 | 1.429  (1.101, 1.854) | 1.442  (1.112, 1.871) | 1.432  (1.103, 1.858) | 1.453  (1.112, 1.897) |
|  | *P* for interaction |  |  |  |  |  | 0.001 | 0.001 | 0.001 | 0.001 |

Note: Model 1, unadjusted model. Model 2, model 1 + adjusted for age and sex. Model 3, model 2 + adjusted for smoking status, alcohol consumption, regular exercise, low income, and obesity. Model 4, model 3 + adjusted for comorbid conditions, including NSAID medication, DM, HTN, DL, and CKD. Abbreviations: CI, confidence interval; CKD, chronic kidney disease; DL, dyslipidemia; DM, diabetes mellitus; HR, hazard ratio; HTN, hypertension; RA, rheumatoid arthritis.

**Supplementary Table 3. Cause-specific Cox regression analysis of RA for the risk of ESRD**

|  | |  | Model 1 | Model 2 | Model 3 | Model 4 |
| --- | --- | --- | --- | --- | --- | --- |
|  |  |  | HR  (95%CIs) | HR  (95%CIs) | HR  (95%CIs) | HR  (95%CIs) |
| Total | | Control | Reference | Reference | Reference | Reference |
|  |  | RA | 2.156  (1.958, 2.374) | 2.157  (1.958, 2.375) | 2.137  (1.940, 2.354) | 2.121  (1.917, 2.347) |
| Age (years) | 20 – 39 | Control | Reference | Reference | Reference | Reference |
|  |  | RA | 8.260  (4.740, 14.394) | 8.261  (4.740, 14.396) | 8.203  (4.707, 14.293) | 7.626  (4.361, 13.332) |
|  | 40 – 64 | Control | Reference | Reference | Reference | Reference |
|  |  | RA | 2.564  (2.238, 2.938) | 2.565  (2.239, 2.939) | 2.533  (2.210, 2.902) | 2.501  (2.175, 2.876) |
|  | 65 ≤ | Control | Reference | Reference | Reference | Reference |
|  |  | RA | 1.651  (1.428, 1.908) | 1.652  (1.429, 1.909) | 1.642  (1.420, 1.898) | 1.656  (1.428, 1.921) |
|  | *P* for interaction |  | < 0.001 | < 0.001 | < 0.001 | < 0.001 |
| Sex | Male | Control | Reference | Reference | Reference | Reference |
|  |  | RA | 1.971  (1.689, 2.299) | 1.972  (1.690, 2.301) | 1.938  (1.660, 2.262) | 1.936  (1.649, 2.274) |
|  | Female | Control | Reference | Reference | Reference | Reference |
|  |  | RA | 2.289  (2.022, 2.591) | 2.289  (2.022, 2.591) | 2.281  (2.015, 2.582) | 2.249  (1.983, 2.550) |
|  | *P* for interaction |  | 0.138 | 0.140 | 0.107 | 0.1427 |
| Smoking status | Former or non-smoker | Control | Reference | Reference | Reference | Reference |
|  |  | RA | 2.117  (1.906,2.351) | 2.114  (1.904,2.348) | 2.097  (1.888,2.329) | 2.081  (1.867, 2.320) |
|  | Current smoker | Control | Reference | Reference | Reference | Reference |
|  |  | RA | 2.411  (1.885,3.084) | 2.425  (1.896,3.102) | 2.377  (1.858,3.041) | 2.372  (1.785, 3.051) |
|  | *P* for interaction |  | 0.340 | 0.315 | 0.359 | 0.3424 |
| Alcohol consumption | No | Control | Reference | Reference | Reference | Reference |
|  |  | RA | 2.011  (1.801, 2.247) | 2.009  (1.798, 2.244) | 2.011  (1.801, 2.247) | 2.000  (1.785, 2.241) |
|  | Yes | Control | Reference | Reference | Reference | Reference |
|  |  | RA | 2.622  (2.152, 3.194) | 2.621  (2.151, 3.193) | 2.618  (2.148, 3.191) | 2.598  (2.121, 3.183) |
|  | *P* for interaction |  | 0.022 | 0.021 | 0.023 | 0.0245 |
| Regular exercise | No | Control | Reference | Reference | Reference | Reference |
|  |  | RA | 2.108  (1.897, 2.344) | 2.108  (1.896, 2.343) | 2.097  (1.886, 2.331) | 2.080  (1.863, 2.322) |
|  | Yes | Control | Reference | Reference | Reference | Reference |
|  |  | RA | 2.382  (1.882, 3.014) | 2.377  (1.879, 3.008) | 2.353  (1.859, 2.978) | 2.347  (1.849, 2.979) |
|  | *P* for interaction |  | 0.354 | 0.361 | 0.382 | 0.3617 |
| Low-income status | No | Control | Reference | Reference | Reference | Reference |
|  |  | RA | 2.164  (1.938, 2.416) | 2.154  (1.929, 2.405) | 2.126  (1.904, 2.374) | 2.101  (1.874, 2.355) |
|  | Yes | Control | Reference | Reference | Reference | Reference |
|  |  | RA | 2.142  (1.754, 2.616) | 2.191  (1.794, 2.676) | 2.174  (1.779, 2.655) | 2.191  (1.788, 2.686) |
|  | *P* for interaction |  | 0.931 | 0.883 | 0.850 | 0.718 |
| Obesity | No | Control | Reference | Reference | Reference | Reference |
|  |  | RA | 2.426  (2.149, 2.740) | 2.438  (2.159, 2.753) | 2.412  (2.135, 2.724) | 2.414  (2.414, 2.737) |
|  | Yes | Control | Reference | Reference | Reference | Reference |
|  |  | RA | 1.784  (1.521, 2.094) | 1.769  (1.507, 2.076) | 1.756  (1.496, 2.061) | 1.725  (1.466, 2.029) |
|  | *P* for interaction |  | 0.003 | 0.002 | 0.002 | 0.0011 |
| DM | No | Control | Reference | Reference | Reference | Reference |
|  |  | RA | 3.243  (2.861, 3.677) | 3.245  (2.862, 3.678) | 3.210  (2.832, 3.640) | 3.131  (2.749, 3.566) |
|  | Yes | Control | Reference | Reference | Reference | Reference |
|  |  | RA | 1.236  (1.053, 1.450) | 1.242  (1.058, 1.457) | 1.231  (1.049, 1.445) | 1.265  (1.076, 1.487) |
|  | *P* for interaction |  | < 0.001 | < 0.001 | < 0.001 | < 0.001 |
| HTN | No | Control | Reference | Reference | Reference | Reference |
|  |  | RA | 5.292  (4.304, 6.507) | 5.302  (4.312, 6.518) | 5.219  (4.245, 6.416) | 5.384  (4.371, 6.633) |
|  | Yes | Control | Reference | Reference | Reference | Reference |
|  |  | RA | 1.619  (1.446, 1.811) | 1.640  (1.466, 1.835) | 1.621  (1.448, 1.814) | 1.670  (1.486, 1.876) |
|  | *P* for interaction |  | < 0.001 | < 0.001 | < 0.001 | < 0.001 |
| DL | No | Control | Reference | Reference | Reference | Reference |
|  |  | RA | 3.151  (2.748, 3.613) | 3.185  (2.778, 3.651) | 3.152  (2.749, 3.613) | 3.199  (2.778, 3.683) |
|  | Yes | Control | Reference | Reference | Reference | Reference |
|  |  | RA | 1.453  (1.264, 1.670) | 1.448  (1.259, 1.664) | 1.436  (1.249, 1.651) | 1.479  (1.283, 1.704) |
|  | *P* for interaction |  | < 0.001 | < 0.001 | < 0.001 | < 0.001 |
| CKD | No | Control | Reference | Reference | Reference | Reference |
|  |  | RA | 3.752  (3.263,4.313) | 3.752  (3.264, 4.314) | 3.715  (3.231, 4.272) | 3.679  (3.187, 4.248) |
|  | Yes | Control | Reference | Reference | Reference | Reference |
|  |  | RA | 1.322  (1.148, 1.523) | 1.328  (1.153, 1.529) | 1.323  (1.149, 1.524) | 1.337  (1.158, 1.543) |
|  | *P* for interaction |  | < 0.001 | < 0.001 | < 0.001 | < 0.001 |

Note: Model 1, unadjusted model. Model 2, model 1 + adjusted for age and sex. Model 3, model 2 + adjusted for smoking status, alcohol consumption, regular exercise, low income, and obesity. Model 4, model 3 + adjusted for comorbid conditions, including NSAID medication, DM, HTN, DL, and CKD. Abbreviations: CI, confidence interval; CKD, chronic kidney disease; DL, dyslipidemia; DM, diabetes mellitus; HR, hazard ratio; HTN, hypertension; RA, rheumatoid arthritis.

**Supplementary Table 4. Propensity score matching analysis for the RA group**

|  | Control (*n* =154997) | RA (*n* =154997) | *P* value | ASD |
| --- | --- | --- | --- | --- |
| Demographic information |  |  |  |  |
| Age (year) | 55.83 ± 11.85 | 55.82 ± 11.90 | 0.718 | 0.001 |
| 20 – 39 | 12972 (8.37) | 13128 (8.47) |  |  |
| 40 – 64 | 104781 (67.6) | 104598 (67.48) |  |  |
| 65 ≤ | 37244 (24.03) | 37271 (24.05) |  |  |
| Male sex | 41533 (26.8) | 41548 (26.81) | 0.952 | 0.000 |
| Current smoking | 17858 (11.52) | 18194 (11.74) | 0.060 | 0.007 |
| Alcohol consumption | 45687 (29.48) | 45824 (29.56) | 0.590 | 0.002 |
| Regular exercise | 28465 (18.36) | 28627 (18.47) | 0.453 | 0.003 |
| Low-income status | 31069 (20.04) | 31309 (20.2) | 0.282 | 0.004 |
| Comorbid conditions |  |  |  |  |
| Use of NSAIDs | 151966 (98.04) | 151966 (98.04) | 1.000 | < 0.001 |
| Obesity | 51693 (33.35) | 51653 (33.33) | 0.879 | 0.001 |
| DM | 19233 (12.41) | 19471 (12.56) | 0.196 | 0.005 |
| HTN | 58547 (37.77) | 58709 (37.88) | 0.549 | 0.002 |
| DL | 52334 (33.76) | 52411 (33.81) | 0.770 | 0.001 |
| CKD | 8758 (5.65) | 9124 (5.89) | 0.005 | 0.010 |
| Anthropometric measures |  |  |  |  |
| Height (cm) | 159.19 ± 8.35 | 159.34 ± 8.35 | < 0.001 | 0.017 |
| Body weight (kg) | 60.7 ± 10.43 | 60.58 ± 10.44 | 0.001 | 0.012 |
| BMI (kg/m^2^) | 23.89 ± 3.23 | 23.8 ± 3.24 | < 0.001 | 0.028 |
| Waist circumference (cm) | 79.86 ± 9.07 | 79.74 ± 9.16 | 0.001 | 0.012 |
| SBP (mmHg) | 122.8 ± 15.34 | 122.06 ± 15.06 | < 0.001 | 0.049 |
| DBP (mmHg) | 75.93 ± 10.02 | 75.5 ± 9.8 | < 0.001 | 0.043 |
| Laboratory findings |  |  |  |  |
| Fasting glucose (mg/dL) | 99.44 ± 23.43 | 98.27 ± 23.01 | <.0001 | 0.050 |
| Total cholesterol (mg/dL) | 198.83 ± 38.42 | 196.24 ± 38.98 | <.0001 | 0.067 |
| eGFR (mL/min./1.73m^2^) | 89.59 ± 39.08 | 90.11 ± 36.99 | 0.000 | 0.014 |

Note: Values for categorical variables are given as number (percentage); values for continuous variables, as mean ± standard deviation. Abbreviations: ASD, absolute standardized mean difference; BMI, body mass index; CKD, chronic kidney disease; DBP, diastolic blood pressure; DL, dyslipidemia; DM, diabetes mellitus; eGFR, estimated glomerular filtration rate; ESRD, end-stage renal disease; HTN, hypertension; NSAIDs, non-steroidal anti-inflammatory drugs; SBP, systolic blood pressure.

**Supplementary Table 5. Cox regression analysis of RA for the risk of ESRD after propensity score matching**

|  | Total number | Event number | Follow-up duration (person-years) | Incidence *per* 1,000  person-years | Crude HR  (95%CIs) | Cause-specific HR  (95%CIs) |
| --- | --- | --- | --- | --- | --- | --- |
| Control | 154,997 | 278 | 735,001.79 | 0.378 | Reference | Reference |
| RA | 154,997 | 590 | 728,414.58 | 0.810 | 2.142  (1.857, 2.470) | 2.130  (1.847, 2.457) |

Note: In the cause-specific hazard model, the death occurring before reaching the primary outcome was treated as a competing risk and censored. Abbreviations: CI, confidence interval; HR, hazard ratio; RA, rheumatoid arthritis.
